# Obesity and HFpEF

**DOI:** 10.3390/jcm11133858

**Published:** 2022-07-03

**Authors:** Francesco Clemenza, Roberto Citarrella, Angelo Patti, Manfredi Rizzo

**Affiliations:** 1 Cardiology Department, IRCCS—ISMETT, 90127 Palermo, Italy; 2 Promise Department, School of Medicine, University of Palermo, 90133 Palermo, Italy

Heart failure with preserved ejection fraction (HFpEF) has represented a therapeutic challenge in recent decades [1], which has only recently begun to find partial (PARAGON) or significant (EMPEROR-Preserved, DELIVER) solutions with randomised clinical trials (RCTs). One of the reasons, probably the main one, for the substantial failure of the RCTs conducted to date lies in the heterogeneity of the clinical/physiopathological pictures included in the umbrella definition of HFpEF [2,3]. This is why the need to design RCTs oriented towards specific phenotypes has long been stressed [4], so as to identify a potentially existing clinical benefit for a given pharmacological treatment in a defined clinical context, a benefit that instead risks being “diluted” if the treatment is applied to a heterogeneous set of clinical and pathophysiological pictures, as happens when the criterion for inclusion in the studies is basically an ejection fraction above a certain cut-off.

From this point of view, an HFpEF phenotype of particular interest is that of obese patients, for several reasons, which are briefly reviewed in the following:

− **Epidemiological:** Cardiovascular diseases are the leading cause of death in obesity [5]; there is an association between body mass index (BMI) and risk of heart failure [6], which—if in the reduced ejection fraction form (HFrEF), may be “masked” by the never definitively clarified “obesity paradox”—is certainly strong and linear in HFpEF. There is also a relationship between obesity and atherosclerotic-based cardiovascular events, which—if not the direct object of this discussion—are certainly related to the prevalence of heart failure in this population.
− **Pathophysiological:** Over the last 10 years, a highly accredited theory has developed that interprets HFpEF as the ultimate consequence of a series of inflammatory mechanisms present in various chronic diseases, in this sense distinguishing it from HFrEF, in which neurohumoral activation mechanisms prevail as the pathogenetic cause of ventricular dysfunction [3,7,8]. In fact, obesity is a chronic inflammatory state, which Schiattarella [9] recently defined as ‘meta-inflammation’, i.e., a chronic low-grade inflammatory response induced by metabolism, which is present in obesity, diabetes and other metabolic diseases, related to the toxic accumulation of lipids (‘lipotoxicity’). Today, it is clear that metabolic disorders, inflammation and impaired cardiac function (pEF) are interconnected, and that obesity represents a clinical/physiopathological model in which this connection is the central element for the development of cardiovascular pathologies, beyond the clinical consequences, although important, intrinsically caused by overweight per se. In this scenario, a particularly important role has been attributed to epicardial fat, as a producer in an autocrine/paracrine mode of inflammatory mediators that act on coronary microcirculation and myocardial cells, promoting the mechanisms leading to HFpEF [10,11]; it has also been shown by various methods (autopsy, CT, and MRI) that epicardial fat is significantly represented in patients with HFpEF [10,12].
− **Clinical:** We have stated that the definition of HFpEF actually includes heterogeneous clinical conditions, which are united by an ejection fraction above a certain cut-off (>50% according to the latest European and American guidelines), but with substantial differences in terms of etiopathogenesis, pathophysiology and clinical presentation. This is one of the reasons, probably the most important one, behind the substantial failure of RCTs on the pharmacological treatment of HFpEF (with the exception of recent studies with SGLT2i), since it is unlikely that a single treatment can provide benefit in such different clinical conditions. Thus, the need arose to identify specific phenotypes, on which to perform RCTs with targeted therapies (in other words, a desirable but not yet realised ‘precision medicine’). There is still no univocally accepted standardisation in the definition of these phenotypes: an attempt—conceptually interesting, but difficult to apply in practice—has been made with the use of ‘big data’, analysing a large number of clinical variables with computer systems, and extrapolating three archetypal models of HFpEF, namely, that of obese, diabetic, and obstructive sleep apnoea patients [13]. More recently, Schiattarella et al. proposed a classification of HFpEF into three phenotypes: the cardiorenal, the autoimmune/inflammatory and the cardiometabolic, characterised precisely by the extensive presence of visceral adipose tissue and epicardial fat [14]. A specificity of the obese phenotype with HFpEF was identified in 2017 by Obokata [15] and in 2018 by Packer [16]; the latter emphasised the unfavourable role played in this context by neprilysin, leptin and aldosterone.
− **Therapeutic perspectives:** On the basis of these observations, Packer hypothesised a possible favourable role of sacubitril/valsartan, MRA and SGLT2i in the obese phenotype with HFpEF [17]; we do not yet have data with RCTs in this specific context, although it cannot be ruled out that the general data of SGLT2i (and to a lesser extent of sacubitril/valsartan) in HFpEF may also have been influenced by these mechanisms; similar considerations could be made for MRAs, which in the TOPCAT study—apart from the known sample bias caused by geographical differences in patient enrolment between America and Eastern Europe—showed a favourable trend compared to the placebo.

A class of drugs that could have a potentially favourable role in the specific phenotype of obese patients with HFpEF is that of the GLP1 RA, which has been demonstrated to effectively reduce body weight, have an effect on epicardial fat [18], a have a documented effect with MRI on visceral fat reduction [19]. In addition to their favourable effects in the anti-atherogenic sense, which underlie their proven efficacy for the prevention of cardiovascular events in the broadest sense, they have an anti-inflammatory action that could be the key to the treatment of HFpEF in the obese phenotype, of which we have already seen how ‘metainflammatory’ mechanisms may accurately represent the pathogenetic basis.

These hypotheses are currently being tested in clinical trials, such as the SELECT [20] RCT enrolling 17,500 patients with BMI > 27 for a semaglutide vs. placebo comparison, with combined primary endpoint cardiovascular deaths + non-fatal myocardial infarction + non-fatal stroke, and the STEP-HFpEF RCT semaglutide vs. placebo, enrolling 516 patients with HFpEF and BMI > 30, with endpoints related to quality of life.

From the outcomes of these studies, we are likely to obtain the answer to the question of the actual role of GLP1 RA in reducing cardiovascular events in obese patients and, in particular, its efficacy in the specific obese phenotype with HFpEF.
